# Serum Albumin Alters [ ^18^ F]FDG Activity in the Liver and Blood Pool

**DOI:** 10.1055/s-0044-1795100

**Published:** 2024-11-19

**Authors:** Wai Ip Li, Kwok Sing Ng, Wai Chung Wong, Koon Kiu Ng, Ting Kun Au Yong, Boom Ting Kung

**Affiliations:** 1 Nuclear Medicine Unit, Department of Diagnostic and Interventional Radiology, Queen Elizabeth Hospital, Yau Ma Tei, Hong Kong

**Keywords:** FDG, PET/CT, biodistribution, albumin, correlation, SUL

## Abstract

**Objective** This study aims to investigate the correlation between the 2-deoxy-2-[ ^18^ F]fluoro-D-glucose ([ ^18^ F]FDG) activity of the liver and blood pool, and the serum albumin.

**Methods** A retrospective analysis was conducted on adult patients who underwent [ ^18^ F]FDG positron emission tomography/computed tomography at the Nuclear Medicine Unit of a hospital in Hong Kong between January 1, 2023, and March 31, 2023. The mean standardized uptake value normalized to lean body mass (SULmean) was measured in the liver and blood pool. Pearson's correlation analyses between the SULmean of reference regions and serum albumin were performed. Multiple linear regression was used to analyze the effects of serum albumin and other parameters as the independent predictors on SULmean of the reference regions.

**Results** A total of 146 patients were included, with their SULmean of the liver and blood pool showing significantly positive correlations with serum albumin ( *r* = 0.393, *p* < 0.001 and *r* = 0.207, *p* = 0.012, respectively). Multiple linear regression analyses confirmed serum albumin as an independent variable on SULmean of the liver and blood pool ( *p* < 0.001 and *p* = 0.014, respectively).

**Conclusion** Serum albumin alters [ ^18^ F]FDG biodistribution in the liver and blood pool. The decrease in liver background activity in patients with low serum albumin may produce a higher false-positive rate of lesion detection, particularly when there is a drop of serum albumin in serial scans. Nuclear medicine physicians should be cautious of image interpretation.

## Introduction

Albumin is the most abundant plasma protein synthesized in the liver, with a concentration ranging from 35 to 50 g/L and a biological half-life of 14 to 20 days. 1 It contributes to 65% of the oncotic pressure and plays an important role in transporting molecules, including hormones, fatty acids, and medications. Hypoalbuminemia, defined as serum albumin concentration below 35 g/L, is a prevalent disorder, particularly among hospitalized, critically ill, and malnourished patients. 2 The pathophysiology of hypoalbuminemia is multifaceted and can result from a combination of different mechanisms. Malnutrition, liver cirrhosis, nephrotic syndrome, protein-losing enteropathy, burns, sepsis, critical illness, and heart failure can result in hypoalbuminemia. 3 4 5 6 7 Serum albumin level is considered a powerful biomarker for disease prognosis and survival in various oncologic diseases, proven useful in esophageal cancer, 8 colorectal cancer, 9 hepatocellular carcinoma, 10 gastric cancer, 11 12 non-small cell lung cancer, 13 14 breast cancer, 15 16 ovarian cancer and primary peritoneal cancer, 17 carcinoma of unknown origin, 18 non-Hodgkin's lymphoma, 19 head and neck squamous cell carcinoma, 20 and renal cell carcinoma. 21 Moreover, hypoalbuminemia is a strong independent prognosticator of nononcologic conditions such as cardiovascular diseases, 22 and it is associated with high mortality in patients with acute illness. 23 In our clinical practice, we have observed altered variations in 2-deoxy-2-[18F]fluoro-D-glucose ([ ^18^ F]FDG) biodistribution, particularly in the liver, among patients with low serum albumin levels. This observation raises concerns about the potential impact on qualitative and quantitative interpretations.

The use of [ ^18^ F]FDG positron emission tomography/computed tomography (PET/CT) has revolutionized noninvasive diagnostic imaging, playing a pivotal role in evaluating a wide range of neoplastic, infective, and inflammatory diseases. While standardized uptake value (SUV) quantitation is commonly utilized to measure metabolic activity in target lesions, visual assessment remains fundamental for qualitative analysis, defining active lesions, and staging and restaging diseases. Typically, liver and blood pool activity serve as qualitative and quantitative uptake references, with higher tissue uptake than the liver indicating genuine lesions. It is well established that certain medical conditions alter [ ^18^ F]FDG biodistribution detected on PET/CT, including the fasting status, hyperglycemia, recent insulin injection, vigorous exercise, hyperbilirubinemia, and liver diseases. 24 25 26

Understanding the correlation between serum albumin and [ ^18^ F]FDG activity in the liver and blood pool, which serve as the metabolic references in disease evaluations, will provide valuable insights into the influence of serum albumin changes on [ ^18^ F]FDG uptake and the altered biodistribution of [ ^18^ F]FDG in individuals with low serum albumin levels. Therefore, the objective of this study is to investigate the correlations of [ ^18^ F]FDG activity in the liver and blood pool with serum albumin levels.

## Methods

### Case Enrollment

Patients who underwent [ ^18^ F]FDG PET/CT at the Nuclear Medicine Unit of a hospital in Hong Kong between January 1, 2023, and March 31, 2023, were retrospectively reviewed. Those with age ≥ 18 years, blood glucose level < 11 mmol/L before [ ^18^ F]FDG injection, and serum albumin measured within 4 weeks of PET/CT examinations were included in this study. We excluded subjects with [ ^18^ F]FDG-avid lesion in the tissue of interest for evaluation and the presence of fatty liver or evidence of cirrhosis on plain CT. Furthermore, we excluded those on renal replacement therapy. Clinical and biochemical parameters were reviewed.

### PET/CT Image Protocol and Acquisition

Patients fasted for at least 4 hours before tracer injection. Intravenous administration of [ ^18^ F]FDG with a mean activity of 393.9 ± 43.9 MBq was given, followed by PET/CT acquisition after a mean uptake interval of 58.2 ± 3.4 minutes. [ ^18^ F]FDG PET/CT scans were performed using a PET/CT system, Discovery 710 (GE Healthcare, Milwaukee, Wisconsin, United States). PET images were acquired using the three-dimensional acquisition mode with a duration of 2 minutes per bed position covering from vertex to mid-thigh. Ordered subset expectation maximization with time-of-flight and point spread function modeling was applied, utilizing 4 iterations and 18 subsets, with a 5.5-mm cutoff filter. Slice thickness was set at 3.27 mm and a matrix size of 256 × 256. CT was acquired using the helical mode with a voltage of 120 kVp and a current ranging from 80 to 400 mA, modulated with a noise index of 15. Rotation speed was set at 0.5 seconds/rotation with a pitch of 0.984. Slice thickness and increment were set at 3.75 and 3.27 mm, respectively. Matrix size was set at 512 × 512.

### Image Analysis and Evaluation of [ ^18^ F]FDG Uptake in the Reference Regions

**Fig. 1:**
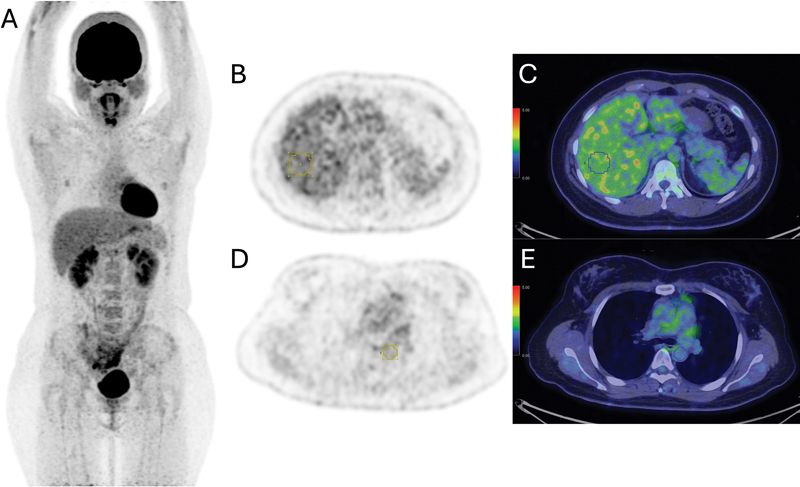
Example of SUL measurement at the reference regions in a patient with normal serum albumin of 41 g/L. ( **A** ) Maximal intensity projection image. ( **B, C** ) Transaxial PET and fusion images showing the measurement of SULmean of the liver using a 3-cm-diameter VOI in the right side of the liver, midway between the dome and inferior margin, excluding the central ducts and vessels. ( **D, E** ) Transaxial PET and fusion images showing the measurement of SULmean of the blood pool using a 1-cm-diameter spherical VOI at the descending thoracic aorta, at the level of the carina. PET, positron emission tomography; SUL, standardized uptake value normalized to lean body mass; SULmean, mean standardized uptake value normalized to lean body mass; VOI, volume of interest.

A nuclear medicine physician with 5 years of experience in molecular imaging evaluated the [ ^18^ F]FDG PET/CT images using the Volume Viewer (Advanced Workstation version 12.3, Ext 8; GE Healthcare). The measurement of SUV in a volume of interest (VOI) was normalized to lean body mass (SUL) using the James formula, to minimize the influence of the amount of body fat. 27 28 The mean of SUL in the VOI (SULmean) was measured in the liver and blood pool, as shown in Fig. 1 . The SULmean of the liver was calculated using a 3-cm-diameter spherical VOI in the right side of the liver, midway between the dome and inferior margin, excluding the central ducts and vessels, as per international standard. 29 The SULmean of the blood pool was calculated using a 1-cm-diameter spherical VOI in the descending thoracic aorta, at the level of the carina.

### Data Analysis

The descriptive statistics of the patients were reported according to their frequencies or means accompanied by standard deviations. Data were tested with Kolmogorov–Smirnov's test for normality. The correlations between the SULmean of reference regions and serum albumin were quantified using the Pearson's correlation analysis. Multiple linear regression analyses were used to analyze the effects of serum albumin and other parameters including the age, blood glucose level, dose of [ ^18^ F]FDG administrated, uptake time, total bilirubin, alkaline phosphatase, alanine transaminase, and serum creatinine as the independent predictors on the SULmean of the liver and blood pool. The statistical analysis was performed using the SPSS Statistics (IBM SPSS Statistics for Macintosh, Version 29.0.1.0. Armonk, New York, United States: IBM Corp). All hypothesis tests were two sided with a *p* -value of < 0.05 considered statistically significant.

## Results

A total of 146 patients, 70 females and 76 males, were included with a mean serum albumin of 37.1 ± 6.5 g/L, with all patients coming for evaluation of malignancies. Patients' demographics, clinical characteristics, and the SULmean of liver and blood pool are shown in Table 1 .

**Table 1:** Patients' demographics, clinical characteristics, and SULmean of reference regions

Features	Frequency or mean ± standard deviation
Number of subjects	146
Gender	
Female	70
Male	76
Age (y)	60.5 ± 16.7
Body mass index (kg/m ^2^ )	22.1 ± 4.0
Blood glucose before [ ^18^ F]FDG injection (mmol/L)	5.9 ± 1.4
Dose of [ ^18^ F]FDG injection administrated (MBq)	393.9 ± 43.9
Uptake interval (min)	58.2 ± 3.4
Serum albumin (g/L)	37.1 ± 6.5
Total bilirubin (μmol/L)	8.2 ± 4.7
Alkaline phosphatase (IU/L)	93.1 ± 41.0
Alanine transaminase (IU/L)	27.2 ± 36.4
Serum creatinine (μmol/L)	77.6 ± 34.8
SULmean of	
Liver	1.85 ± 0.31
Blood pool	1.34 ± 0.23

Abbreviations: [ ^18^ F]FDG, 2-deoxy-2-[18F]fluoro-D-glucose; SULmean, mean standardized uptake value normalized to lean body mass.

**Fig. 2:**
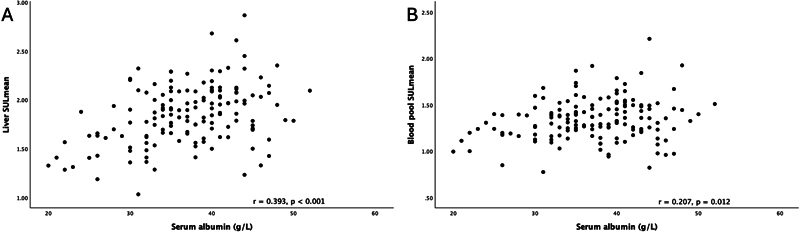
( **A** ) Scatter plot of SULmean of the liver against serum albumin showed a significantly positive correlation ( *r* = 0.393, *p* < 0.001). ( **B** ) Scatter plot of SULmean of the blood pool against serum albumin showed a significantly positive correlation ( *r* = 0.207, *p* = 0.012). SULmean, mean standardized uptake value normalized to lean body mass.

Scatter plots of SULmean in the reference regions against serum albumin are shown in Fig. 2 with results of Pearson's correlation analyses ( *r* as correlation coefficient). The SULmean of the liver showed a significantly positive correlation with the serum albumin ( Fig. 2A , *r* = 0.393, *p* < 0.001), and the SULmean of the blood pool showed a significantly positive correlation with the serum albumin ( Fig. 2B , *r* = 0.207, *p* = 0.012).

Multiple linear regression analyses were used to analyze the effects of serum albumin, age, blood glucose level, dose of [ ^18^ F]FDG administrated, uptake interval, total bilirubin, alkaline phosphatase, alanine transaminase, and serum creatinine on the SULmean of the liver and blood pool, and results are illustrated in Tables 2 and 3 , respectively. Serum albumin was identified to be a significant independent variable that predicted the SULmean of the liver ( Table 2 , β = 0.356, *p* < 0.001) and blood pool ( Table 3 , β = 0.220, *p* = 0.014), with the greatest standardized β coefficients in the liver SULmean. Other significant independent variables that influenced SULmean of the reference regions were shown in the tables.

**Table 2:** Multiple regression analysis using dependent variable of liver SULmean

Parameters	Unstandardized β coefficient	Coefficient standard error	Standardized β coefficient	*p* -Value
Serum albumin	0.017	0.004	0.356	**< 0.001**
Age	0.001	0.002	0.029	0.730
Blood glucose before [ ^18^ F]FDG injection	0.024	0.017	0.109	0.164
Dose of [ ^18^ F]FDG injection administrated	0.001	0.001	−0.006	0.940
Uptake interval	−0.013	0.007	−0.146	**0.050**
Total bilirubin	−0.001	0.005	−0.010	0.901
Alkaline phosphatase	−0.002	0.001	−0.227	**0.012**
Alanine transaminase	0.002	0.001	0.294	**< 0.001**
Serum creatinine	0.001	0.001	0.129	0.091

Abbreviations: [ ^18^ F]FDG, 2-deoxy-2-[18F]fluoro-D-glucose; SULmean, mean standardized uptake value normalized to lean body mass.

Note: Bold indicates statistically significant results ( *p* < 0.05).

**Table 3:** Multiple regression analysis using dependent variable of blood pool SULmean

Parameters	Unstandardized β coefficient	Coefficient standard error	Standardized β coefficient	*p* -Value
Serum albumin	0.008	0.003	0.220	**0.014**
Age	0.001	0.001	0.099	0.258
Blood glucose before [ ^18^ F]FDG injection	0.017	0.013	0.105	0.196
Dose of [ ^18^ F]FDG injection administrated	0.001	0.001	−0.025	0.751
Uptake interval	−0.015	0.005	−0.218	**0.005**
Total bilirubin	−0.004	0.004	−0.073	0.363
Alkaline phosphatase	−0.001	0.001	−0.215	**0.022**
Alanine transaminase	0.001	0.001	0.184	**0.041**
Serum creatinine	0.002	0.001	0.241	**0.003**

Abbreviations: [ ^18^ F]FDG, 2-deoxy-2-[18F]fluoro-D-glucose; SULmean, mean standardized uptake value normalized to lean body mass.

Note: Bold indicates statistically significant results ( *p* < 0.05).

## Discussion

We aimed to investigate the correlation of [ ^18^ F]FDG activity in the liver and blood pool with serum albumin levels. According to the literature search, this is the first study using SUL, which minimizes the influences of SUV measurement by the proportions of body fat and lean tissue. 27 Our findings revealed that there was a significantly positive correlation between the SULmean of the liver and serum albumin ( *p* < 0.001) and a significantly positive correlation between the SULmean of the blood pool and serum albumin ( *p* = 0.012), which were consistent with a prior study. 30 Serum albumin was also shown to be the independent variable that predicted the SULmean of the liver and blood pool ( *p* < 0.001 and *p* = 0.014, respectively). Our research suggests a potential relationship between [ ^18^ F]FDG biodistribution and serum albumin, in which the underlying mechanism, however, remains poorly understood, and this topic has not been extensively discussed in the literature to date. Otomi et al suggested that with decreased albumin synthesis from the liver, the hepatic energy demand and thus the glucose consumption decreased, which explained the decreased [ ^18^ F]FDG activity in the liver. 30 Further investigations are needed to explain these findings with evidence.

We performed multiple linear regression analyses to assess the effects of serum albumin and other relevant parameters on SULmean of the liver and blood pool. The liver and mediastinal blood pool activity were known to be decreasing continuously with time after administration of [ ^18^ F]FDG, 31 in consistence with our results. The liver enzymes had shown to be independent variables on SULmean of the liver and blood pool. Elevated liver enzymes were multifactorial, and they might be indicative of fatty liver disease which was correlated with background liver uptake intensity, 24 26 and thus, it might alter biodistribution of [ ^18^ F]FDG as well. PET and CT were not sensitive for early changes of liver diseases in spite of our exclusion criteria. Yet, there was no large size study to investigate the relationship between liver enzymes and [ ^18^ F]FDG biodistribution. Serum creatinine level was shown to be a significant independent variable on the blood pool SULmean, which was evidenced by a previous literature focusing on the relationship of liver and blood pool [ ^18^ F]FDG uptake with the estimated glomerular filtration rate, which revealed their negative correlations. 32 On the contrary, serum creatinine showed no significant correlation with the liver uptake in this study, potentially due to our included subjects with normal or slightly abnormal renal function only. Age had shown to be positively correlated with liver activity in research, 33 but there was no correlation with the liver SUL in our results, which could be due to the difference of normalization methods of SUV and the high mean age of our study.

The application of [ ^18^ F]FDG activity in the liver as the qualitative and quantitative references for the assessment of various diseases is widely accepted, as recommended by international guidelines. Examples include the Deauville score for high-grade lymphoma 34 and multiple myeloma, 35 Hopkins criteria and Cuneo score in head and neck squamous cell carcinoma, 36 treatment response assessment in solid malignancies using Positron Emission Tomography Response Criteria in Solid Tumors (PERCIST), 29 and evaluation of large vessel vasculitis and polymyalgia rheumatica. 37 While a genuine lesion is commonly defined as target tissue with activity higher than that of the liver, the lower liver background activity in patients with low serum albumin may cause a higher lesion-to-liver uptake ratio and false-positive results. This can lead to erroneous upstaging of diseases, potentially resulting in suboptimal treatments. Therefore, caution must be exercised when interpreting [ ^18^ F]FDG PET/CT scans, particularly in cases where there is a drop in serum albumin between the serial scans. Using the mediastinal blood pool activity as the metabolic reference was suggested due to its less significant correlation to serum albumin. 30 In addition, given the positive correlation between the liver [ ^18^ F]FDG activity and serum albumin, [ ^18^ F]FDG uptake of the liver may serve as a potentially independent prognostic factor for various benign and malignant conditions, reflecting nutritional status in a manner similar to the serum albumin. Future prospective studies with larger and more diverse cohorts are warranted to validate these findings and explore the underlying mechanisms in greater detail.

This study had several limitations. First, this was a single-center retrospective study. The study population consisted of patients with advanced diseases, leading to a relatively high prevalence of hypoalbuminemia (32.9%, 48 out of 146). Our study was based mainly on Chinese subjects with high mean age. Therefore, the generalizability of the findings and the applicability of the results to general populations and other ethnicities might be limited. Also, it is important to note that although patients with liver lesions detected on imaging were excluded from the study, [ ^18^ F]FDG PET/CT is generally less sensitive in detecting primary liver lesions, which might impact the measurement of liver activity and potentially affect the interpretation of the results.

## Conclusion

Serum albumin levels are positively correlated with [ ^18^ F]FDG activity in the liver and blood pool. It is an independent variable on the liver and blood pool [ ^18^ F]FDG activity. Further prospective studies with larger and more diverse cohorts are warranted to validate the clinical applications. Nuclear medicine physicians should be aware of the factors contributing to altered biodistribution in [ ^18^ F]FDG PET/CT and exercise caution when interpreting images.
